# Alterations of the Ceramide Metabolism in the Peri-Infarct Cortex Are Independent of the Sphingomyelinase Pathway and Not Influenced by the Acid Sphingomyelinase Inhibitor Fluoxetine

**DOI:** 10.1155/2015/503079

**Published:** 2015-10-28

**Authors:** R. Brunkhorst, F. Friedlaender, N. Ferreirós, S. Schwalm, A. Koch, G. Grammatikos, S. Toennes, C. Foerch, J. Pfeilschifter, W. Pfeilschifter

**Affiliations:** ^1^Department of Neurology, Goethe University, 60590 Frankfurt am Main, Germany; ^2^Institute of General Pharmacology and Toxicology, Goethe University, 60590 Frankfurt am Main, Germany; ^3^Institute of Clinical Pharmacology, Goethe University, 60590 Frankfurt am Main, Germany; ^4^Department of Internal Medicine I, Goethe University, 60590 Frankfurt am Main, Germany; ^5^Institute of Forensic Medicine, Goethe University, 60590 Frankfurt am Main, Germany

## Abstract

Ceramides induce important intracellular signaling pathways, modulating proliferation, migration, apoptosis, and inflammation. However, the relevance of the ceramide metabolism in the reconvalescence phase after stroke is unclear. Besides its well-known property as a selective serotonin reuptake inhibitor, fluoxetine has been reported to inhibit the acid sphingomyelinase (ASM), a key regulator of ceramide levels which derives ceramide from sphingomyelin. Furthermore, fluoxetine has shown therapeutic potential in a randomized controlled rehabilitation trial in stroke patients. Our aim was to investigate and modulate ceramide concentrations in the peri-infarct cortex, whose morphological and functional properties correlate with long-term functional outcome in stroke. We show that certain ceramide species are modulated after experimental stroke and that these changes do not result from alterations of ASM activity, but rather from nontranscriptional induction of the ceramide *de novo* pathway. Unexpectedly, although reducing lesion size, fluoxetine did not improve functional outcome in our model and had no significant influence on ASM activity or the concentration of ceramides. The ceramide metabolism could emerge as a potential therapeutic target in the reconvalescence phase after stroke, as its accumulation in the peri-infarct cortex potentially influences membrane functions as well as signaling events in the tissue essential for neurological recovery.

## 1. Introduction

Stroke is a disease of enormous socioeconomic relevance. Worldwide, it is the second leading cause of death and the leading cause of adult disability [1]. So far, acute stroke therapies aim at recanalizing the occluded brain vessels by means of pharmacologic thrombolysis or thrombectomy. Decades of research on (neuro)protective drugs have established several promising candidates that reduced infarct size in experimental animal models of stroke with positive effects on neurological outcome in short term observations. However, none of these substances could prove efficiency in large scale randomized controlled trials in stroke patients. A relatively new experimental approach is to target the dysfunction and mechanism of recovery within the nonischemic tissue surrounding the infarcted area, the so-called peri-infarct cortex. The peri-infarct tissue of the photothrombotic stroke model shows high morphological similarities to the penumbra in other stroke models such as distal middle cerebral artery occlusion, but most importantly, this model allows the investigation of long-term functional outcome in mice [2].

Sphingolipids are a complex class of signaling molecules and an essential part of cellular membranes. Their cellular levels regulate proliferation, apoptosis, and inflammation depending on the specific sphingolipid species, cell and receptor type, and different intracellular targets [3]. Ceramides are the backbone of more complex sphingolipids and the precursor of the versatile signaling molecule sphingosine-1-phosphate. They are essential for specific membrane functions (e.g., the formation of lipid rafts and caveolae) [3] and directly modulate intracellular effector proteins such as PKC*ζ* [4], c-Raf [5], and CAPP [6]. Ceramide is tightly regulated in the cells, and its participation in cell death signaling pathways is controlled by rapid conversion of ceramide into less noxious/toxic sphingolipids. Ceramides are generated in different cellular compartments by three different pathways (Supplemental Figure 3 in Supplementary Material available online at http://dx.doi.org/10.1155/2015/503079): the* de novo* pathway in the endoplasmatic reticulum, the salvage/sphingomyelinase pathway in the Golgi, lysosome, and the plasma membrane, as well as by recycling of glycosphingolipids. The cellular function of ceramides is in part dependent on the chain length, which is determined by differential activity of specific ceramide synthases (CerS 1–6) [7].

There is some evidence that alterations in sphingolipid metabolism, leading to enhanced ceramide production, occur in neurological disorders, such as multiple sclerosis [8], Wilson's disease [9], and Alzheimer's disease [10]. Cytokines such as tumor necrosis factor-alpha (TNF-*α*) [11] and reactive oxygen species (ROS) [12] induce the production of ceramide through activation of sphingomyelinases in this context. Therefore, the activation of the sphingomyelinase pathway is believed to be a general cellular stress response.

In experimental stroke, there are several studies showing an increase in ceramide synthesis via higher acid sphingomyelinase (ASM) activity [13–15] directly within the ischemic lesion. However, these studies did not investigate the relevance for long-term functional outcome as they focused on the ischemic, death prone tissue and a short observation period. In that context, an experimental reduction of ceramide production was shown to be (neuro)protective leading to smaller infarct sizes and better short term outcome.

Intriguingly, the antidepressant fluoxetine, a selective serotonin reuptake inhibitor and the only drug which has been shown to improve poststroke motor recovery in a randomized controlled neurorehabilitation trial in stroke patients [16], has recently been shown to inhibit ASM activity in the rodent healthy brain [17]. As the different functions of ceramide indicate a possible role in peri-infarct inflammatory, degenerative, and recovery processes, we hypothesized that ceramides are relevant for long-term functional outcome after stroke. Given the potential of fluoxetine to inhibit the ASM, the prorehabilitative effect of fluoxetine in stroke recovery might be attributable to the modulation of ceramide levels in the peri-infarct cortex.

We therefore hypothesized that (a) the peri-infarct region shows alterations of ceramide levels, that (b) these alterations result from a change of ASM activity, and (c) that fluoxetine improves motor recovery after stroke, and this effect might be mediated by ASM inhibition.

## 2. Methods

### 2.1. Photothrombotic Stroke and Drug Administration and Behavioral Testing

All animal experiments were approved by the local government authorities (Regierungspraesidium Darmstadt). Sample size calculations for all experiments were performed, assuming a power of 80%, previously published standard deviations and a difference between treatment groups of 20%. Photothrombosis (PT) was performed as previously described [18]. Briefly, 6–8 week old C57/Bl6 mice were anaesthetized by 1.7% isoflurane and 0.1 mg/kg buprenorphine s.c. and placed into a stereotactical frame. Five minutes after injection of 0.2 mL rose-bengal i.p. (Sigma-Aldrich, Taufkirchen, Germany; 10 mg/mL), the skull was illuminated at the motor cortex by a cold light source. At days 1, 3, 7, and 28, respectively, 10 mice per group (sham, saline, and fluoxetine) were killed by a lethal dose of isoflurane and immediate transcardial perfusion with 0.9% saline. After measurement of stroke size by either TTC staining or a photograph of the brain surface, 10 mg blocks of the peri-infarct cortex (Supplemental Figure 1) were dissected and immediately frozen in liquid nitrogen. At days −7, 7, and 28, behavioral outcome was determined by the cylinder task and the grid-walking test as previously described [19]. The observer was blinded for treatment groups. Fluoxetine (ratiopharm, Ulm, Germany) was given from day 3 to day 28 after stroke via drinking water (concentration: 120 mg/L) as previously published [17]. Fluoxetine plasma-levels were measured at day 7 and 28 (Supplemental Method 1).

### 2.2. LC-MS/MS

For quantification of ceramides, their precursors and metabolites, about 10 mg tissue was homogenized with PBS and liquid-liquid extracted with methanol : chloroform : HCl (15 : 83 : 2). The analytical procedure was similar to the method published elsewhere ([20] see Supplemental Method 2).

### 2.3. Sphingomyelinase Activity

The samples were lysed in 250 mM sodium acetate (pH 5.0), 1% NP40 and 1.3 mM EDTA. The tissue was then homogenized using the TissueLyser LT (1 min, 50 Hz; Qiagen, Hilden, Germany). Aliquots of the lysates were diluted to 250 mM sodium acetate (pH 5.0), 0.1% NP40, and 1.3 mM EDTA, then incubated with 10 nCi per sample [^14^C]sphingomyelin for 10 min at 37°C. The reaction was stopped by addition of 600 *μ*L of chloroform/methanol (2 : 1), and phases were separated by centrifugation. Radioactivity of the aqueous phase was quantified by scintillation counting and enabled quantification of ASM activity. For the NSM assay another lysis-buffer (HEPES 100 mM; 0.1% NP40; 5 mM DTT; 10 mM MgCL_2_; 1.4 mM EDTA), reaction, and suspension buffer (HEPES 100 mM; 0.1% NP40; 5 mM DTT; 10 mM MgCL_2_) were used. Substrate concentration, protein concentration, and reaction time were chosen after pilot studies (Supplemental Figures 2C and D).

### 2.4. RT-PCR

After homogenization using the TissueLyser LT (1 min, 50 Hz; Qiagen, Hilden, Germany), 1.2 *µ*g of total RNA was isolated with TRIZOL (Sigma-Aldrich, Steinheim, Germany) according to the manufacture's protocol and used for reverse transcriptase-polymerase chain reaction (RT-PCR; Revert Aid first strand cDNA synthesis kit, Thermo Fisher Scientific, St. Leon-Rot. Germany) utilizing an oligo (dT) primer for amplification.

Real-time PCR (TaqMan) was performed using Applied Biosystems 7500 Fast Real-Time PCR System. Probes, primers, and the reporter dyes 6-FAM and VIC were from Life Technologies (Darmstadt, Germany). The cycling conditions were as follows: 95°C for 15 min (1 cycle), 95°C for 15 s, and 60°C for 1 min (40 cycles). The threshold cycle (C_t_) was calculated by the instrument software (7500 Fast System SDS Software version 1.4). Analysis of the relative mRNA expression was performed using the ΔΔC_t_ method. The housekeeping gene GAPDH was used for normalization.

## 3. Results

**Figure 1:**
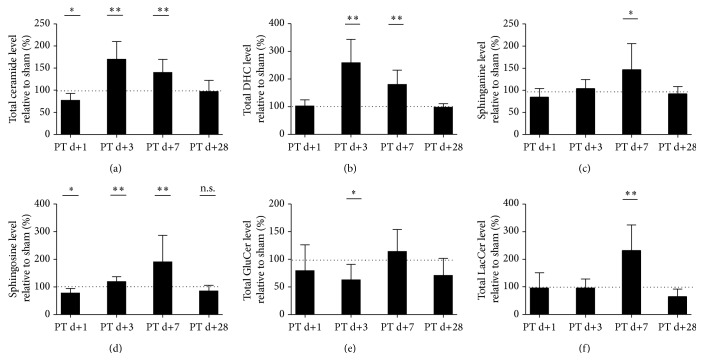
Ceramide, its precursors, and its derivate levels are altered in the peri-infarct cortex after photothrombotic stroke. (a) Total ceramide levels, (b) dihydroceramide levels, (c) sphinganine levels, (d) sphingosine levels, (e) glucosylceramide levels, and (f) lactosylceramide levels. Sphingolipids were measured at the indicated time points by tandem mass spectrometry. Differences between sham and PT group were analyzed using Student's unpaired two-tailed *t*-test. Data are presented as means ± SD; sham values are indicated by dotted line (for individual sham-SD's, see Section 3); values are not significantly different compared to sham if not marked otherwise; ^*∗*^ *p* ≤ 0.05; ^*∗∗*^ *p* ≤ 0.01; n.s., nonsignificant; *n* = 8–10/group.

Mass spectrometry revealed a reduction of total ceramide levels in the peri-infarct cortex (Figure 1(a)) at day 1 after photothrombosis (77.19% ± 15.51) compared to the corresponding cortex area in sham-operated mice (100% ± 21.5, *p* = 0.02, *n* = 8–10). However, at day 3 we found a significant increase of total ceramide (170% ± 39.79 versus 100% ± 24.85, *p* = 0.0003, *n* = 8–10), which persisted up to day 7 (140% ± 29.58 versus 100% ± 23.8, *p* = 0.0035, *n* = 10). Interestingly, the direct ceramide precursor of the* de novo* pathway, dihydroceramide (DHC, Figure 1(b), Supplemental Figure 3), was correspondingly increased at day 3 (258.5% ± 85 versus 100% ± 14.74, *p* < 0.0001, *n* = 8–10) and day 7 (180% ± 52 versus 100% ± 12.4, *p* = 0.0002, *n* = 10). Both ceramide and DHC normalized compared to sham at day 28. The precursor of DHC is sphinganine (Figure 1(c)), which was increased at day 7 after photothrombosis (147% ± 58.62 versus 100% ± 19.12, *p* = 0.0274, *n* = 10). Sphingosine (Figure 1(d)), the precursor as well as derivate of ceramide via the CerS or the ceramidases, but present at much lower concentrations than ceramide [3], was found to be decreased at day 1 (78% ± 16.46 versus 100% ± 16.26, *p* = 0.0115, *n* = 8–10) but increased at day 3 (119% ± 17.47 versus 100% ± 9.4, *n* = 8–10, *p* = 0.0083) and day 7 (191% ± 95.89 versus 100% ± 12.3, *p* = 0.0083, *n* = 10). Next we checked for an effect of stroke on the glycosphingolipid metabolites. Total glucosylceramides (Figure 1(e)), which can be both a precursor of ceramide via the glucocerebrosidase (GBA) and a product of the glucosylceramide synthase, was reduced at day 3 (63% ± 28.21 versus 100% ± 29.09, *n* = 8–10, *p* = 0.0146). Total lactosylceramides however (Figure 1(f)) were elevated at day 7 (232% ± 92.48 versus 100% ± 36.05, *n* = 10, *p* = 0.0005).

**Figure 2:**
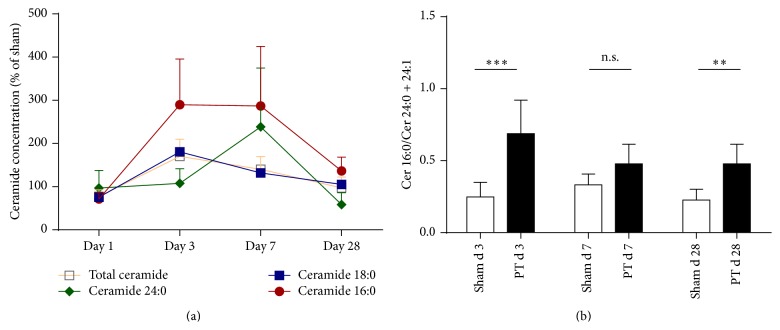
Ceramide subspecies are differentially regulated in the peri-infarct cortex. (a) Total ceramide, ceramide 16:0, 18:0, and 24:0 in the time course after photothrombotic stroke compared to sham. Asterisks for *p* values as well as ceramide 18:1 and 24:1 are not shown (see Section 3). (b) Ratios for ceramide 16:0/ceramide 24:0 + 24:1. Data are presented as means ± SD. Differences between sham and PT group were analyzed using Student's unpaired two-tailed *t*-test.^*∗∗*^ *p* ≤ 0.01; ^*∗∗∗*^ *p* ≤ 0.001; n.s., nonsignificant; *n* = 8–10.

In the peri-infarct cortex, ceramide subspecies levels were differentially changed (Figure 2). Ceramide 18:0 is by far the most abundant ceramide species in the CNS [21] and contributed as the major part of the here observed ceramide elevation. Therefore, its dynamic reproduces that of total ceramides. It was reduced at day 1 compared to sham (Figure 2(a), 76% ± 18.06 versus 100% ± 23.59, *p* = 0.0285, *n* = 8–10) and then increased at day 3 (181% ± 46.97 versus 100% ± 24.68, *p* = 0.0009, *n* = 8–10). Ceramide 16:0 was decreased at day 1 (72% ± 15.33 versus 100% ± 23.52, *p* = 0.0096) then increased at day 3 (290% ± 106 versus 100% ± 29.02, *p* < 0.0001, *n* = 8–10) and day 7, but normalized at day 28. Ceramide 18:1 and ceramide 20:0 behaved in similar fashion (data not shown). The very-long chain ceramides 24:0 and 24:1 however increased only at a later time point at day 7 (238.7% ± 136.1 versus 100% ± 15.71, *p* = 0.0053, *n* = 8–10, ceramide 24:0). All measured ceramide subspecies concentrations returned to baseline at day 28. This resulted in changes of the ratios of ceramides with long chain length to ceramides with very-long chain length (ceramide 16:0/ceramide 24:0 + ceramide 24:1, Figure 2(b)) compared between day 3 and day 7 (0.69 ± 0.23 versus 0.47 ± 0.74, ANOVA, mean difference 0.2095, 95% CI of diff. 0.01397 to 0.4051, *n* = 8–10).

**Figure 3:**
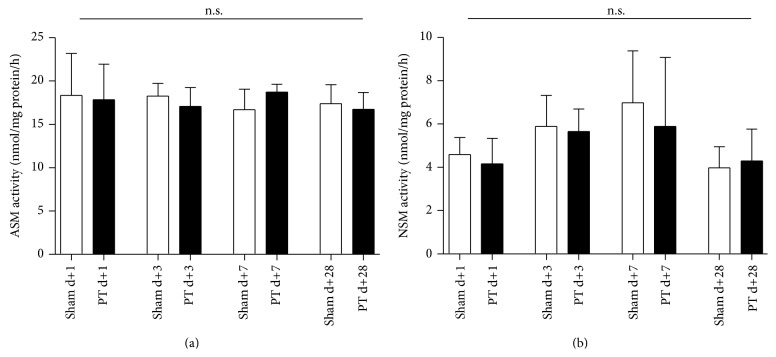
Sphingomyelinase activity is not altered in the peri-infarct cortex. (a) ASM activity and (b) NSM activity. Sphingomyelinase activity was measured at different time points by an enzyme activity assay with radioactive labeled sphingomyelin. Differences between sham and PT group were analyzed using Student's unpaired two-tailed *t*-test. Data are presented as means ± SD; n.s., nonsignificant; *n* = 5–10/group.

As our hypothesis was relying on the assumption that the sphingomyelinase pathway is upregulated under proinflammatory and hypoxic conditions, we measured sphingomyelinase activity in the peri-infarct cortex. However, the activity of both ASM and the NSM were unchanged (Figure 3); ASM activity was higher compared to NSM activity.

**Figure 4:**
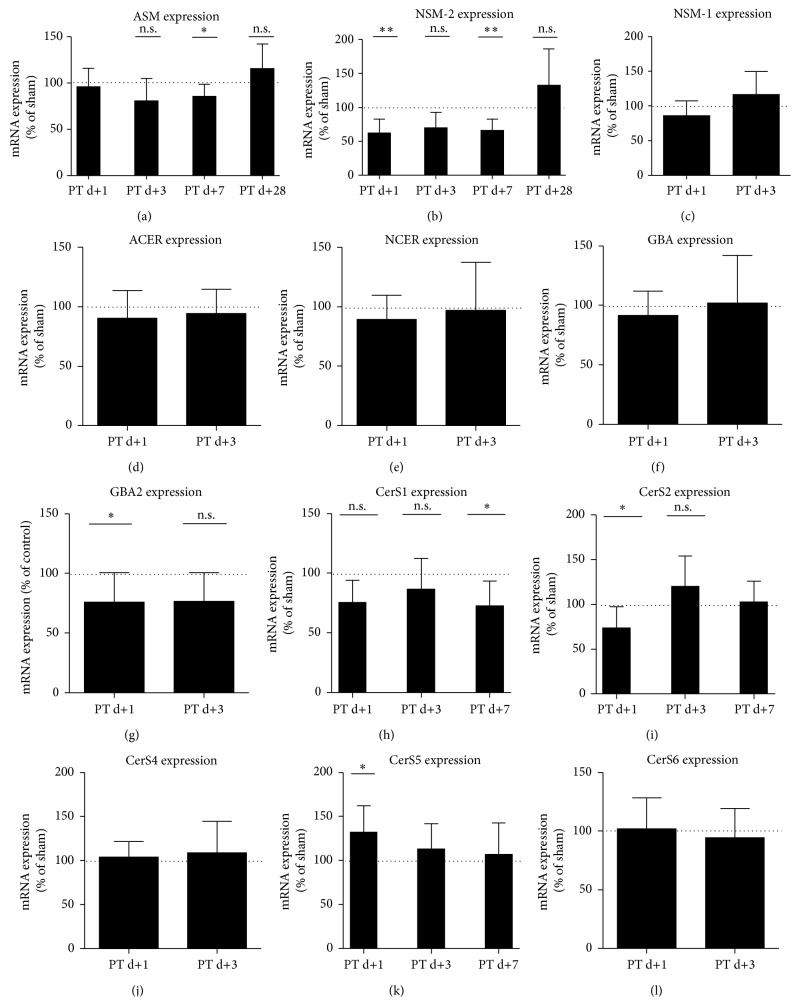
Expression of ceramide-metabolizing enzymes is differentially regulated in the peri-infarct cortex. (a) ASM mRNA, (b) neutral sphingomyelinase- (NSM-) 2 mRNA, (c) NSM-1 mRNA, (d) Acid ceramidase (ACER) mRNA, (e) neutral ceramidase (NCER) mRNA, (f) glucocerebrosidase- (GBA-) 1 mRNA, (g) GBA-2 mRNA, and (h)–(l) CerS1–6 mRNA, mRNA-levels were measured at the indicated time points by Taqman-PCR. Differences between sham and PT group were analyzed using Student's unpaired two-tailed *t*-test. Data are presented as means ± SD; sham values are indicated by dotted line (for individual sham-SD's, see Section 3); values are not significantly different compared to sham if not marked otherwise; ^*∗*^ *p* ≤ 0.05; ^*∗∗*^ *p* ≤ 0.01; n.s., nonsignificant; *n* = 8–10/group.

In order to investigate a potential transcriptional regulation of the different ceramide generation pathways, we performed Taqman PCR of the most relevant enzymes (Supplemental Figure 3). Against our hypothesis but consistent with enzyme activity, we found a reduction of ASM mRNA (Figure 4(a)) at day 7 (85% ± 13.21 versus 100% ± 12.73, *n* = 8–10, *p* = 0.0312), and of the neutral sphingomyelinase 2 (NSM-2, Figure 4(b)) at day 3 (62% ± 20.12 versus 100% ± 21.88, *n* = 9-10, *p* = 0.0012) and day 7 (66% ± 16.77 versus 100% ± 24.82, *n* = 9-10, *p* = 0.0026). NSM-1 mRNA (Figure 4(c)), which is present in much lower quantities than the NSM-2 mRNA (data not shown) was unchanged, as well as the acid ceramidase (ACER) mRNA (Figure 4(d)), the neutral ceramidase (NCER) mRNA (Figure 4(e)), and the acid glucocerebrosidase 1 (GBA1) mRNA (Figure 4(f)). GBA2 mRNA (Figure 4(g)) was reduced at day 1 (76% ± 25 versus 100% ± 17.59, *n* = 9, *p* = 0.0297). CerS1 (Figure 4(h)) is mainly responsible for the* de novo* synthesis of ceramide 18:0 and 20:0 and is downregulated at day 7 (66% ± 20.73 versus 100% ± 25.65, *n* = 9, *p* = 0.026). CerS2 (Figure 4(i)) produces ceramide 20:0 to 26:0 and is downregulated at day 1 (74% ± 23.65 versus 100% ± 24.41, *n* = 9, *p* = 0.0335). CerS4 (responsible for ceramide 18:0 and 20:0) and CerS6 (ceramide 14:0, 16:0 and 18:0) were unchanged (Figures 4(j) and 4(l)), whereas CerS5 (ceramide 16:0) was upregulated at day 1 (132% ± 30.12 versus 100% ± 15.71, *n* = 9, *p* = 0.0120).

**Figure 5:**
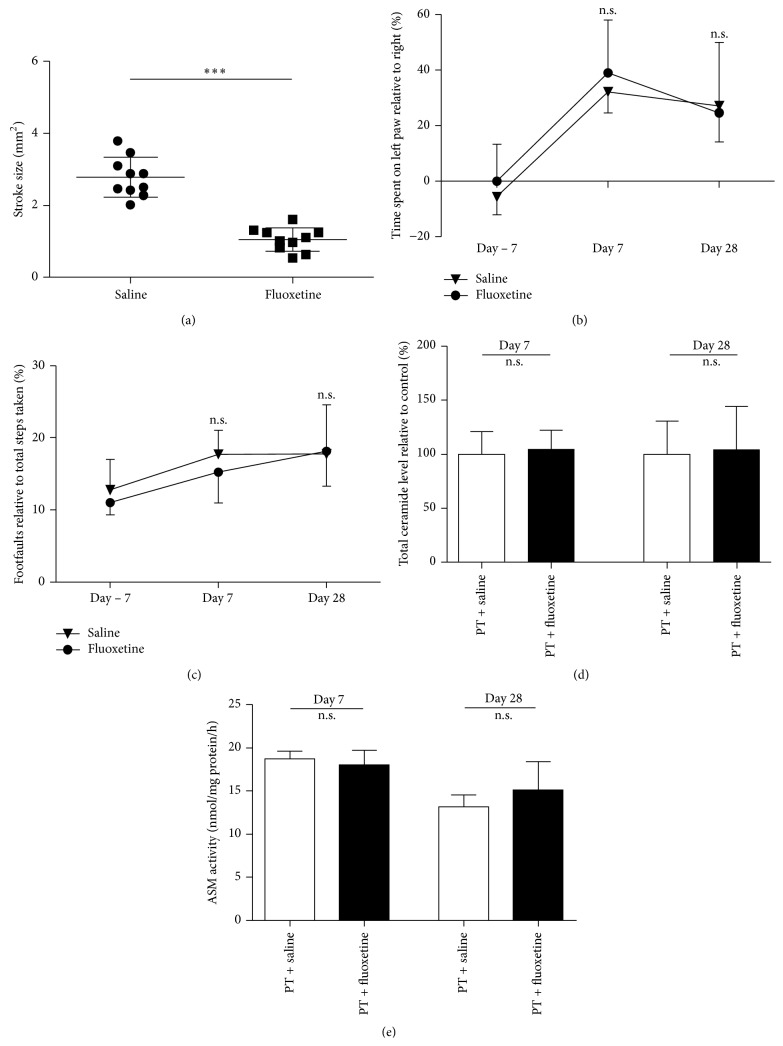
Fluoxetine treatment from day 3 to day 28 reduces infarct size, but has no impact on functional outcome, ASM activity or ceramide levels in the peri-infarct cortex. (a) Stroke size at day 28, (b) functional outcome, measured by the cylinder test, (c) functional outcome, measured by the forelimb grid-walking test, (d) total ceramide levels, and (e) ASM activity. Stroke size was determined by measuring the infarcted cortical area, ceramide level, ASM activity was measured at different time points as indicated above. Differences between saline and fluoxetine were analyzed using Student's unpaired two-tailed *t*-test. Data are presented as means ± SD; ^*∗∗∗*^ *p* ≤ 0.001; n.s., nonsignificant; (a), (d), and (e): *n* = 5–10/group; (b) and (c): *n* = 10/group.

Nevertheless we anticipated a reduction of ceramide levels by inhibiting the acid sphingomyelinase pathway by treatment with the functional inhibitor fluoxetine, starting at day 3 after photothrombosis. We found an effect of fluoxetine on stroke/glial scar size at day 28 (1.047 mm^2^ ± 0.33 versus 2.778 mm^2^ ± 0.55, *p* < 0.0001, *n* = 10; Figure 5(a)) but could not observe any effect of fluoxetine on total ceramide levels (Figure 5(d)) and ASM activity (Figure 5(e)) and most importantly on functional outcome (Figure 5(b)). As controls for the validity of our assay as well as the potency of fluoxetine and other antidepressants to inhibit the ASM at the concentrations we achieved in our mice, we measured ASM activity in vitro (Supplemental Figure 2A) and cortex of ASM homozygous and heterozygous knockout mice (Supplemental Figure 2B); in all cases, a reduction of ASM activity could be observed.

## 4. Discussion

The present study evaluated whether targeting ceramide-metabolism might be of potential therapeutic value in the poststroke reconvalescence period. We show a reduction of the bioactive sphingolipid ceramide in the subacute phase (day 1, Figure 1(a)) and an increase of ceramide in an intermediate time-window (day 3–7) within the peri-infarct region. Importantly, in this model, fluoxetine is not able to reduce sphingomyelinase activity, ceramide concentrations or to improve functional outcome. In order to determine other potential therapeutic targets, we investigated the ceramide synthesis pathways in the peri-infarct cortex and could show that the sphingomyelinase is not the responsible enzyme for the ceramide elevations but possibly changes in ceramide* de novo* synthesis.

This study has some major differences to previous studies, describing ceramides in experimental stroke, as these studies only investigated ceramide-levels in the infarct zone [13–15]. In ischemic tissue, the proapoptotic mechanism of increased sphingomyelinase activity has not only been shown in CNS, but also in various other cell types [22]. However, in the here investigated photothrombotic peri-infarct zone, apoptosis only takes place in a fine boundary zone and a short time window [23]. It is therefore unlikely that the elevation of ceramides we observed here, simply reflects mechanisms of cell death, especially as we surprisingly observed a decrease of total ceramide levels at 24 h post stroke, when relatively late penumbral apoptosis is supposed to be present and show only an increase from day 3 to day 7 after stroke. Diffusion from the ischemic core is rather unlikely due to ceramide's poor solubility. Importantly, we did not find any relevant changes of the acid and neutral sphingomyelinase activity (Figure 4) in the peri-infarct tissue compared to sham operated mice. Again, this is not comparable to previous data in experimental stroke, in which the induction of ASM-activity was a very early and short-term phenomenon [13–15]. In summary, we did not detect the very early increase of ASM-activity seen by others probably due to the analysis of different tissue and other time points.

Interestingly, although we found effects of late-onset fluoxetine treatment on infarct size and/or scar formation (Figure 5(a)), which is consistent with previous publications [24, 25], we did not see any effects of fluoxetine on sphingomyelinase activity, ceramide, or behavioral outcome (Figures 5(b)–5(e)). As this finding is a discrepancy to the previously reported effect of fluoxetine on ASM activity in C57/Bl6 mice [17], we performed various controls and could show the inhibition of ASM by fluoxetine in cell cultures, as well as a reduced ASM-activity in ASM homozygous knock-out animals (Supplemental Figure 2), validating the specificity and sensitivity of our assay. Furthermore, the plasma levels of the very lipophilic and blood-brain barrier penetrable drug fluoxetine were fairly high (day 7: 2.25 *µ*M; day 28: 3.76 *µ*M, *n* = 9-10). Unfortunately, we are not able to supply any well-known positive control of fluoxetine action in vivo, such as serotonin reuptake inhibition. However, the effect of fluoxetine on infarct size indicates that fluoxetine mediated some protective effect within the CNS. Additionally, our result is line with others, who could not show an effect of long-term treatment of mice with fluoxetine on ceramides in the CNS [26] and with previous data showing no effect of fluoxetine on recovery after stroke in rats [27, 28].

Our analysis included the direct precursors and derivate of ceramide (Supplemental Figure 3), allowing conclusions to be drawn to the involvement of other ceramide synthesis pathways. Exceptions are ceramide-1-phosphate, which is present at a much lower quantity than ceramide and sphingomyelin, whose potential turnover was quantified by sphingomyelinase activity assays. The here observed increase of dihydroceramides (DHC) (Figure 1(b)) might indicate that instead of the sphingomyelinase pathway, the ceramide* de novo* pathway is upregulated within the peri-infarct tissue. Within this pathway, dihydroceramide (DHC) is the direct precursor of ceramide but also has biological signaling functions on its own [29] and importantly is not a derivate of ceramide. The serine-palmitoyl-Coa transferase (SPT) and the dihydroceramide desaturase (DES), both enzymes of the* de novo* pathway, are regulated by oxidative stress, hypoxia, and inflammation [30, 31]. However, the SPT is less likely to be the only responsible enzyme in the peri-infarct tissue, as we observed only an effect of stroke on sphinganine concentrations on day 7 (Figure 1(c)). As we show here, a parallel increase of ceramide and DHCs, the upregulation of ceramide synthesis probably involves enzymes more proximal than the DES. The enzymes distal of the SPT and proximal of the DES in the* de novo* pathway and responsible for synthesis of ceramide subspecies with specific chain lengths are the ceramide synthases [32]. Considering that our results show that an involvement of the SPT and the DES is less likely and that the increase of certain ceramide subspecies is responsible for the elevation of total ceramide, it is tempting to speculate that the ceramide synthases are responsible for the here observed elevation of ceramides. On the transcriptional level, most of the enzymes of the different ceramide synthesis pathways appear not to contribute to increased ceramide levels (Figure 4); only the increase of CerS5 mRNA (Figure 4(k)) at day 1 could explain the upregulation of Ceramide 16:0 ceramide at day 3–7. Furthermore, the decrease of CerS2 (Figure 4(i)), the second most common CerS in the CNS [33], could contribute to the decrease of total ceramide at day 1. However, it is important to consider, that discrepancies of ceramide synthase activity and transcriptional regulation has been shown previously [33, 34].

In the peri-infarct cortex, instead of cell death, events such as axonal sprouting, microglial and astrocytic activation, angio-/neurogenesis, and synaptic plasticity determine neurological outcome [2]. What could be the pathophysiological role of the observed increase of total ceramide and ceramide subspecies in the peri-infarct cortex? One has to keep in mind that most of the previous data about the effect of ceramide on CNS cells was determined in cell culture using short chain ceramide (Ceramide 2:0–6:0), which has very different properties compared to the more abundant long or very-long ceramides (Ceramide 16:0–24:0; [7]).

Concerning neuronal plasticity, PKC*ζ* can be recruited and activated by ceramide [4, 35] and PKC*ζ* has been shown to be important for long-term potentiation (LTP) creation and maintenance [36]. Ceramide-controlled lipid rafts are essential for efficient synaptic transmission, supporting an influence of ceramide levels on synaptic plasticity; for example, it has been shown that increasing ceramide levels increase NMDA receptor-mediated synaptic transmission [37, 38]. The increase of NMDA-receptor transmission facilitates long-term-potentiation and memory consolidation but on the other hand has a negative impact in the long-term by increasing excitotoxicity. In the case of recovery from experimental stroke, increased glutamatergic transmission in the peri-infarct cortex might be of advantage [19]. On the other hand, there is a negative influence of ceramide on axonal outgrowth, both by ceramide itself [39] and its metabolites [40]. Importantly, a recent publication shows that lactosylceramide controls astrocytic activation in an autocrine fashion, which indirectly influences microglial activity [41]. As we observe an increase of lactosylceramide at day 7 (Figure 1(f)), lactosylceramide may also play a role in (micro)glial activation in the peri-infarct cortex. In multiple sclerosis plaques, total ceramide has been shown to be reduced, but certain subspecies were increased within reactive astrocytes, contributing to blood-brain barrier damage [8]. Ceramide is an inhibitor of both neuro- and angiogenesis [17, 42], two important mechanisms for recovery after stroke [2]. Additionally, a more general neurotoxic effect of ceramide 16:0 and ceramide 24:0 could be recently shown [43].

There is some evidence that specific alterations of different ceramide subspecies occur in CNS diseases such as Alzheimer's disease [44]. This indicates that ceramides substantially influence pathophysiological processes in a manner dependent on the chain length. Concerning membrane functions, ceramide 16:0 and ceramide 24:0 have a different ability to form cluster with cholesterol [45], which is an essential step towards lipid raft formation [46] and clustering as well as activation of various proteins at the cell membrane [47]. Membranes of Cers2-KO-mice (with a lack of ceramide 24:0) are more fluid [48], which indicates that our data could have implications not only for cell membrane function, but also for the functionality of membrane bearing organelles in cells of the peri-infarct cortex. In the peri-infarct cortex we observed a differential regulation of ceramides with specific chain lengths (Figure 2), indicating a specific pathophysiological role for each ceramide subspecies. It appears as if the ceramide metabolism compensates the initially increased ratio of long to very long chain length at day 3 by upregulation of ceramide 24:0 and 24:1 leading to a transient, almost normalized ratio at day 7 (Figure 2(b)).

In summary, our observation of alterations of ceramide, its subspecies and metabolites in the peri-infarct cortex could have both a positive and a negative impact on recovery and function of the peri-infarct tissue after stroke. Unfortunately, the relevance of our observations remains to be proven, as we were not able to reduce ceramide generation by inhibition of sphingomyelinase activity. Future studies should address the potential targets pointed out in this study, that is, the ceramide* de novo* pathway, by other genetic and pharmacological approaches.

## 5. Conclusion

Our observation of an elevation of ceramide points to a complex, but well-coordinated pathophysiological role of sphingolipid metabolism in the peri-infarct cortex. This could have implications for our understanding of recovery from stroke and other acute CNS diseases and indicates potential therapeutic targets.

## Supplementary Material

Supplemental Methods 1 and 2 describe the mass-spectrometry methods for measuring sphingolipids as well as fluoxetine. Supplemental Figure 1 shows the tissue used for biochemical analysis, Supplemental Figure 2 shows the applied control experiments for validation of the ASM-Assay, Supplemental Figure 3 displays the most important lipids and enzymes of the ceramide metabolism.
