# Supplementary material for: Alterations of the Ceramide Metabolism in the Peri-Infarct Cortex Are Independent of the Sphingomyelinase Pathway and Not Influenced by the Acid Sphingomyelinase Inhibitor Fluoxetine
Source: Neural Plast. 2015 Oct 28;2015:503079. doi: 10.1155/2015/503079 (PMC4641186; doi:10.1155/2015/503079)
Supplement: Supplementary file 1 — Supplemental Methods 1 and 2 describe the mass-spectrometry methods for measuring sphingolipids as well as fluoxetine. Supplemental Figure 1 shows the tissue used for biochemical analysis, Supplemental Figure 2 shows the applied control experiments for validation of the ASM-Assay, Supplemental Figure 3 displays the most important lipids and enzymes of the ceramide metabolism. [file 503079.f1.zip › Supplementary methods.docx]

Supplemental Method 1: **Measurement of fluoxetine levels**

Fluoxetine was determined using a downscale of an established liquid chromatography-mass spectrometry (1100 LC series coupled via electrospray interface to a time of flight-mass spectrometer, both from Agilent, Waldbronn, Germany) method which is regularly verified in proficiency tests for therapeutic drug monitoring. Briefly, 20 µl sample were diluted with 20 µl 1 M diammonium hydrogenphosphate buffer pH 9.5 and 50 µl of internal standard solution (1 ng/µl fluoxetine-d6 in acetonitrile) followed by liquid-liquid extraction using 1 ml of 1-chlorobutane:diethylether (1:1, v/v) which was evaporated to dryness at 25°C. The dry residue was reconstituted in 100 µl of 0.1% formic acid:acetonitrile (80:20, v/v) of which 2 µl were injected into the chromatographic system. Calibration of fluoxetine in serum was performed in the range of 0.25 to 2.5 mg/l and 3 quality control levels were used.

Supplemental Method 2: **Measurement of sphingolipid levels**

For quantitation of sphingolipids, 20 µL serum were extracted with methanol:chloroform:HCl (15:83:2) . Afterwards amounts of sphingolipids and internal standards were analyzed by liquid chromatography coupled to tandem mass spectrometry (LC-MS/MS). A Luna C18 column (150 mm x 2 mm ID, 5 µm particle size, 100 Å pore size; Phenomenex, Aschaffenburg, Germany) was used for chromatographic separation. The HPLC mobile phases consisted of water-formic acid (100:0.1, v/v) (A) and acetonitrile-tetrahydrofuran-formic acid (50:50:0.1, v/v/v) (B). For separation, a gradient program was used at a flow rate of 0.3 ml/min. The initial buffer composition 60 % (A)/40 % (B) was hold for 0.6 min and then in 3.9 min linearly changed to 0 % (A)/100 % (B) and hold for 6.5 min. Subsequently the composition was linearly changed within 0.5 min to 60% (A)/40 % (B) and then held for another 4.5 min. The running time for every sample (injection volume: 15 µl) was 16 min. MS/MS analyses were performed on a QTrap 4000 equipped with an APCI (Atmospheric Pressure Chemical Ionization) ion source (AB Sciex, Darmstadt, Germany). The analysis was done in Multiple Reaction Monitoring (MRM) mode. For every analyte two transitions were recorded: one for quantification and another one for qualification, to exclude false positive results, with a dwell time of 50 ms. Analyst Software 1.5 (AB Sciex, Darmstadt, Germany) was used for analysis and quantification. Linearity of the calibration curve was proven for the the sphingolipids of interest. The coefficient of correlation was at least 0.99. Variations in accuracy were less than 15 % over the whole range of calibration.
