# Supplementary figures and images for: Alterations of the Ceramide Metabolism in the Peri-Infarct Cortex Are Independent of the Sphingomyelinase Pathway and Not Influenced by the Acid Sphingomyelinase Inhibitor Fluoxetine
Source: Neural Plast. 2015 Oct 28;2015:503079. doi: 10.1155/2015/503079 (PMC4641186; doi:10.1155/2015/503079)

A

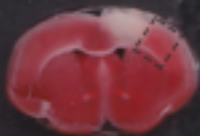

B

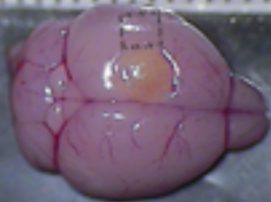

Supplement: Supplementary file 1 — Supplemental Methods 1 and 2 describe the mass-spectrometry methods for measuring sphingolipids as well as fluoxetine. Supplemental Figure 1 shows the tissue used for biochemical analysis, Supplemental Figure 2 shows the applied control experiments for validation of the ASM-Assay, Supplemental Figure 3 displays the most important lipids and enzymes of the ceramide metabolism. [file 503079.f1.zip › Supplemental_Figure_1_NP_1357980.pdf]

**A**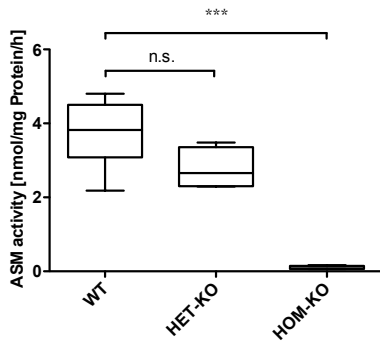**B**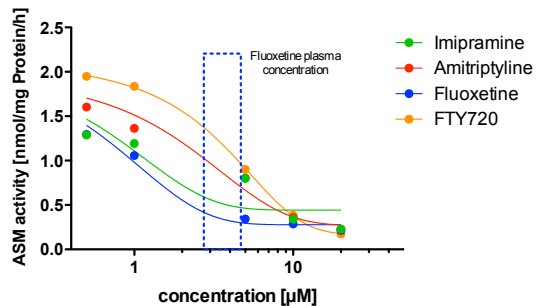**C**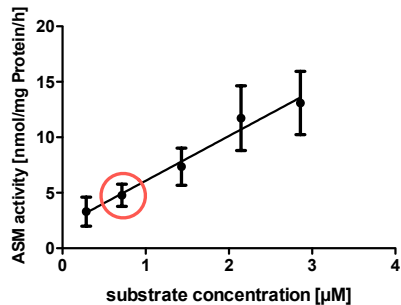**D**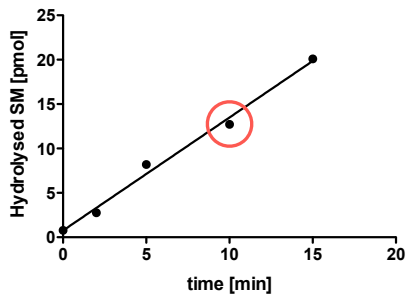

Supplement: Supplementary file 1 — Supplemental Methods 1 and 2 describe the mass-spectrometry methods for measuring sphingolipids as well as fluoxetine. Supplemental Figure 1 shows the tissue used for biochemical analysis, Supplemental Figure 2 shows the applied control experiments for validation of the ASM-Assay, Supplemental Figure 3 displays the most important lipids and enzymes of the ceramide metabolism. [file 503079.f1.zip › Supplemental_Figure_2_NP_1357981.pdf]

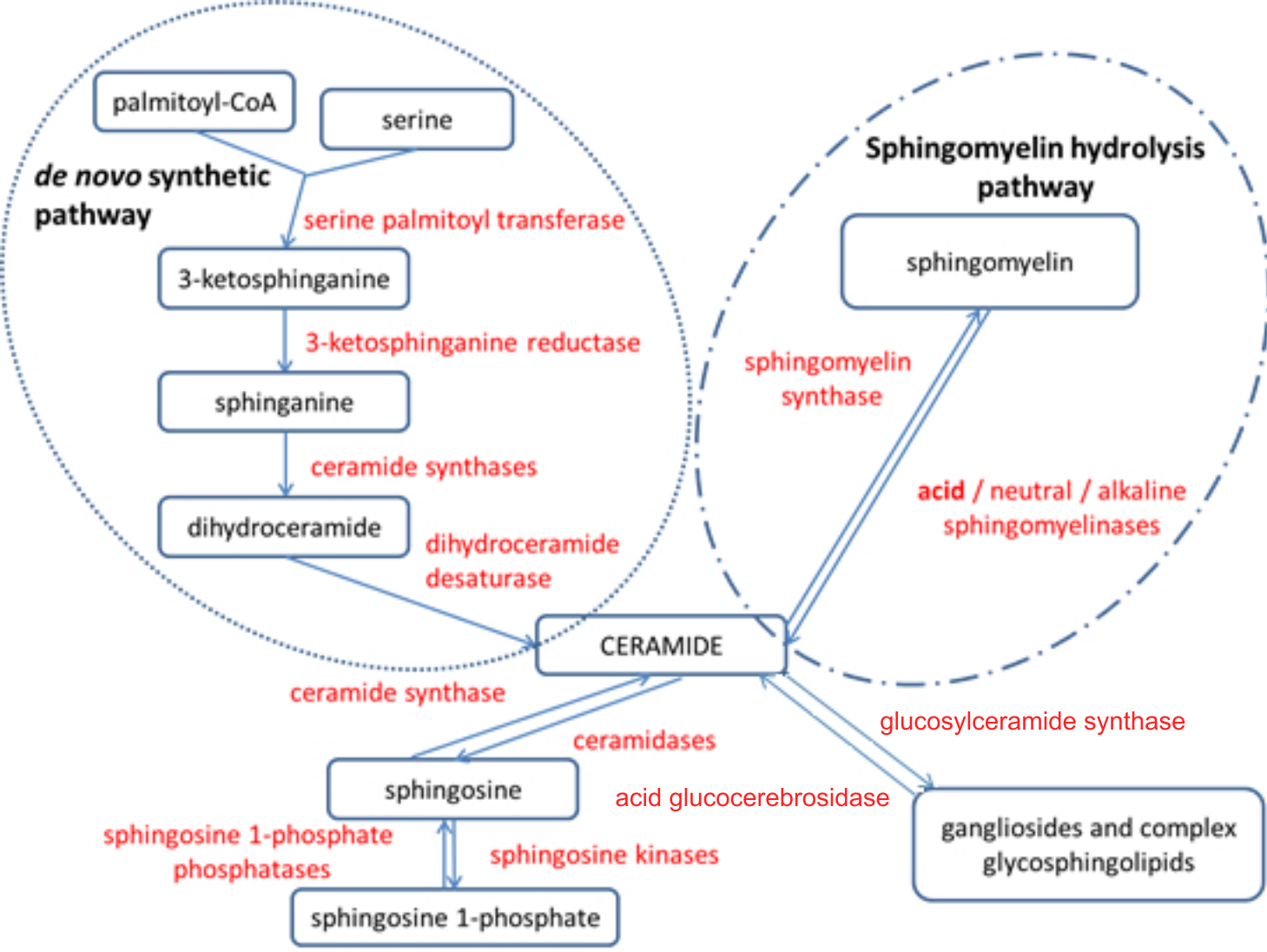

Supplement: Supplementary file 1 — Supplemental Methods 1 and 2 describe the mass-spectrometry methods for measuring sphingolipids as well as fluoxetine. Supplemental Figure 1 shows the tissue used for biochemical analysis, Supplemental Figure 2 shows the applied control experiments for validation of the ASM-Assay, Supplemental Figure 3 displays the most important lipids and enzymes of the ceramide metabolism. [file 503079.f1.zip › Supplemental_Figure_3_NP_1357982.pdf]
